# The Materiobiology of Silk: Exploring the Biophysical Influence of Silk Biomaterials on Directing Cellular Behaviors

**DOI:** 10.3389/fbioe.2021.697981

**Published:** 2021-06-22

**Authors:** Dakshi Kochhar, Megan K. DeBari, Rosalyn D. Abbott

**Affiliations:** ^1^Department of Biomedical Engineering, Carnegie Mellon University, Pittsburgh, PA, United States; ^2^Department of Materials Science and Engineering, Carnegie Mellon University, Pittsburgh, PA, United States

**Keywords:** silk, biomaterials, tissue engineering, materiobiology, cellular behaviors

## Abstract

Biophysical properties of the extracellular environment dynamically regulate cellular fates. In this review, we highlight silk, an indispensable polymeric biomaterial, owing to its unique mechanical properties, bioactive component sequestration, degradability, well-defined architectures, and biocompatibility that can regulate temporospatial biochemical and biophysical responses. We explore how the materiobiology of silks, both mulberry and non-mulberry based, affect cell behaviors including cell adhesion, cell proliferation, cell migration, and cell differentiation. Keeping in mind the novel biophysical properties of silk in film, fiber, or sponge forms, coupled with facile chemical decoration, and its ability to match functional requirements for specific tissues, we survey the influence of composition, mechanical properties, topography, and 3D geometry in unlocking the body’s inherent regenerative potential.

## Introduction

To form and regenerate tissues, cells attain a staggering amount of molecular information from their microenvironment; where the extracellular matrix (ECM) is not only a “guiding” element for cells, but also highly responsive to cellular behavior (Place et al., 2009). The goal of tissue engineering is to provide cues that stimulate these extraordinary native processes to engineer lost or damaged tissue. Toward this goal, instructive and dynamic features in scaffolds (Li et al., 2017) can be used to drive the body’s intrinsic organizational potential and self-repair abilities.

Biomaterials are never truly inert, being at best biotolerable. The cell-substrate interface serves as more than just a boundary separating the host and material; instead, it introduces physical and chemical cues for cellular adhesion and the subsequent induction of tissue generation or rejection (Biggs et al., 2010). Chemical constituents have been the focus of biomaterial design for several years, but there is increasing recognition of the significance of other material features such as mechanical properties, topology, and 3D geometry in directing cellular behavior (Cukierman et al., 2001; Stevens and George, 2005; Curtis et al., 2006). The ECM’s stiffness can independently dictate differentiation into cells as functionally divergent as bone and nerve (Pelham and Wang, 1997; Engler et al., 2006). The topography and hydrophilicity of biomaterials can enable cellular adhesion even in the absence of cell adhesion peptides (Woo et al., 2003). Dynamic tuning of material properties including availability of cellular adhesion sequences (Kloxin et al., 2009), mechanical characteristics (Guvendiren and Burdick, 2012) and ECM degradability (Khetan et al., 2013) can induce changes in cellular behavior. Such emerging dynamic biomaterial chemistries can provide a “give and take” between cells and materials (Murphy et al., 2014). This concept has been termed “materiobiology” and was introduced by Li et al. (2017) to describe the influence of materials on biological functions at different cellular levels.

**FIGURE 1:**
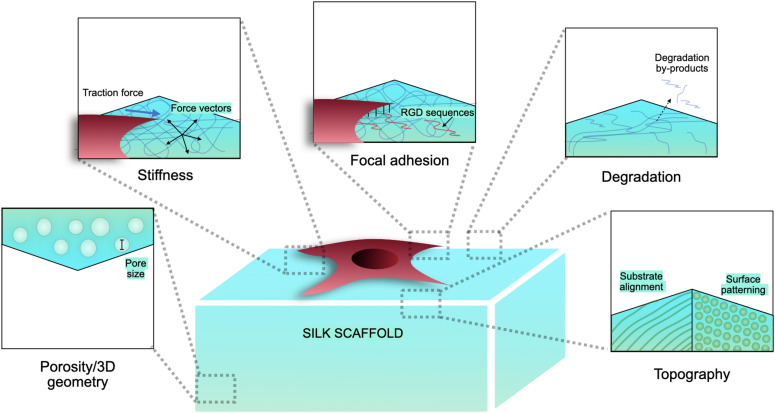
Biophysical characteristics of silk scaffolds.

**FIGURE 2:**
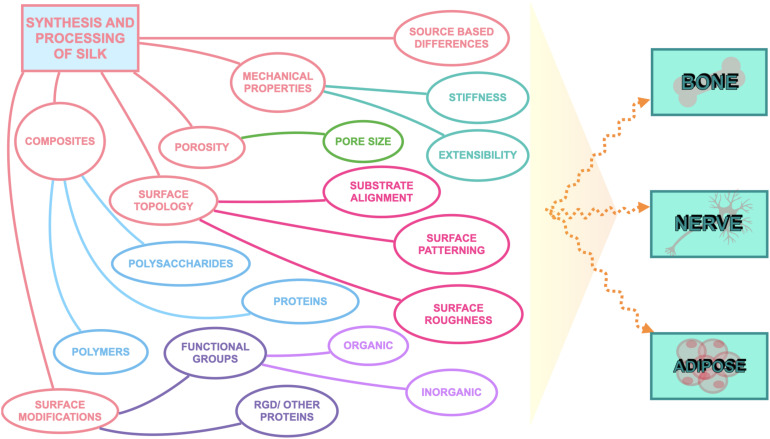
Considerations to tailor silk biomaterials to guide cell fate.

Silk has emerged as a natural biomaterial that can govern, and perhaps even trigger, specific stem cell differentiation based on its intrinsic toughness, mechanical strength, biocompatibility, molecular tunability, topography, geometry, chemical functionality, degradability, and degradation by-products (Figure 1; Altman et al., 2003; Karageorgiou et al., 2006; Pritchard et al., 2011). Silks are spun into fibrous polymers by certain lepidopteral larvae like silkworms, spiders, scorpions, and flies. Silk fibroin (SF), a fibrous protein derived from *B. mori*, will be the primary focus of this review given its extensive use in tissue regeneration (Kaplan et al., 1993; Altman et al., 2003). On the basis of feeding habitats, silkworm-based silk can be broadly classified as mulberry (*B. mori* from Bombycidae family) and non-mulberry (Saturniidae family) (Kaplan et al., 1993). While silks vary in structure, composition, and features based on their source, silks are characterized by highly repetitive primary sequences that contribute to homogeneity in their β-sheet secondary structure. In contrast with globular proteins, the β-sheet structure of silk displays superior mechanical properties that are highly tunable. For instance, silk scaffolds can withstand high compressive loads without failure for bone tissue engineering applications (Cunniff et al., 1994). Whereas for ligaments, the high tensile strength of silk biomaterials can be used to reinstate knee function (Altman et al., 2002). This review discusses the materiobiology of silk, highlighting its ECM-mimicking potential and application in stimulating tissue regeneration (Table 1) by influencing cellular adhesion, proliferation, migration, and differentiation. Specifically, materiobiology design considerations will be addressed for tailoring cellular fate: topology (alignment, patterning, roughness), surface modifications, composites, mechanical properties, and material source (Figure 2).

**TABLE 1:** Influence of materiobiological cues on tissue differentiations.

Tissue	Silk form/composition	Cell type	Material property category	Materiobiological cue	Extra points	References
Bone	Co^2+^-doped HA/SF	Adipose-derived mesenchymal stem cells (ASCs)	Surface modification	Co^2+^-doped calcium phosphate (CaP) functionalization	Silk can replace the organic phase of bone matrix (collagen 1)	Fani et al., 2019
	poly(D,L-lactic acid)/SF	Rat osteoblasts	Surface modification	Surface functionalization using SF, variation of hydrophilicity		Cai et al., 2002
	BMP-2 loaded SF	Human bone marrow derived mesenchymal stem cells (hMSCs)	Surface modification	BMP-2 loaded in porous SF scaffolds		Karageorgiou et al., 2006
	SF	ASCs	Surface modification	Functionalization with hydroxyapatite		Ko et al., 2018
	β-sheet-rich silk nanofibers	Bone marrow MSCs	Mechanical properties, Surface topology	Anisotropic morphologies and higher stiffness of 120 kPa		Ding et al., 2020
	Electrospun blend of polylactic acid (PLA) and Tussah SF	Mouse mesenchymal stem cells	Mechanical properties	Young’s modulus 417.65 MPa and tensile strength 180.36 MPa	Tussah silk fibroin (TSF) is rich in Ala, Asp, Arg, and Arg-Gly-Asp (RGD), a motif that promotes cell adhesion	Shao et al., 2016
	SF film	hMSCs	Surface topology	Surface patterning, alignment		Tien et al., 2012
	Nanofibrous A. mylitta silk- poly(caprolactone)	Bone defect models in white New Zealand rabbits	Surface modification, surface topology	High hydrophilicity, high percentage of nitrogen on the surface, and high surface roughness	The repeat sequences of glycine and alanine in natural fibroin are conducive to a rapid β-sheet transformation and can a play a role similar to that of Type I collagen in bone tissue, by acting as nucleation points for HAp	Bhattacharjee et al., 2016
	SF/carboxymethyl cellulose composite nanofibrous scaffold	hMSCs	Substrate alignment	Hydrophilicity, scaffold closely resembles the nanofibrous structure of natural extracellular matrix		Singh et al., 2016
	SF/Decellularized pulp/Collagen/Fibronectin	MG-63	Surface modification, substrate alignment	Biofunctionality, porous fibrillar network		Sangkert et al., 2016
	Diopside/SF nanocomposite	MC3T3-E1	Mechanical properties, porosity	Increased wettability, suitable porosity, high mechanical strength		Ghorbanian et al., 2013
	SF–chitosan/Nano ZrO_2_	Human osteoblast cells	Mechanical properties, porosity	Interconnected porous structure, optimal compressive strength		Teimouri et al., 2015
	SF	hMSCs	Mechanical properties	Slow degradability		Meinel et al., 2005
	Hydroxyapatite-SF	hMSCs	Surface modification	Hydroxyapatite provided nucleation sites for new mineral resulting in the connectivity of trabecular-like architecture		Bhumiratana et al., 2011
	Hydroxyapatite embedded in A. assama silk	hMSCs	Mechanical properties, porosity	High compressive modulus, high porosity, slow degradation rate		Gupta et al., 2016
	Si- and Zn-doped brushite cement/SF	Volumetric femur defects in rabbits	Porosity	Open porous network		Moses et al., 2019
	Diazonium coupled SF	hMSCs	Surface modification	Hydrophilicity		Murphy et al., 2008
	SF	hMSCs	Surface modification	MMP and integrin responses for degradation		Sengupta et al., 2010
	Silk/clay composite	hMSCs	Silk composite	Controlled degradation	Controlling the amount of β-sheets and tuning the secondary structure can provide control over the degradation rate	Mieszawska et al., 2011
	SF	Lewis rats	Silk composite	Controlled degradation		Wang et al., 2008
	Silk-BMP-2	hMSCs	Porosity	Interconnected porous system		Li et al., 2006
Nerve	SF	Human neural stem cells	Surface modification	Short peptide IKVAV linkage		Sun et al., 2017
	SF-graphene hydrogels	Schwann cells	Surface modification, surface alignment, mechanical properties	Bioactive graphene, nanofibrous structure, aligned topography, and mechanical stiffness	Strong synergistic action used through the combination of different cues	Wang et al., 2019 Silk–graphene hybrid hydrogels
	SF film	Neural stem cells	Surface modification	Decoration with integrin-binding laminin peptide motifs (YIGSR and GYIGSR)		Manchineella et al., 2016 Surface-functionalized silk fibroin films
	Poly(lactic-co-glycolic acid)/multi-walled carbon nanotubes/SF	Human adipose tissue-derived stem cells	Substrate alignment, surface modification	Aligned surface topography, entrapped catalpol		Guo et al., 2015
	Golden-Yellow strain *B. mori*	Primary neurons	Biophysical attributes of silk type	Golden-Yellow *B. mori* extracts	Primary neurons cultured on yellow SF displayed a threefold higher neurite length than those grown of white SF films	Pistone et al., 2016
	SF	Neural stem cell	Mechanical properties, substrate alignment	Stiffness similar to nerve tissues, nanofiber topology	Without the influence of specific differentiation biochemical factors	Bai et al., 2014
	SF	Embryonic chick dorsal root ganglion explant culture	Mechanical properties	Permissive mechanical environment for neuronal extension		Hopkins et al., 2013
	SF	Glial RT4-D6P2T cells, adult female Wistar rats	Mechanical properties, porosity	High compressive strength, porous network		Alessandrino et al., 2019
Adipose	SF	hMSCs and hASCs	Mechanical properties, porosity	Macroporosity, long term structural integrity		Mauney et al., 2007
	SF-chitosan	hASCs	Mechanical properties, porosity	Optimal elastic modulus (higher than glass-seeded control), porosity, 3D infiltration, structural integrity		Altman et al., 2010
	SF	hASCs	Mechanical properties	Slow degradation, structural integrity		Bellas et al., 2013
	SF	Rat bone marrow cells	Porosity	Optimal porosity, structural integrity		Mandal and Kundu, 2009b
	SF	Subcutaneous adipose tissue	Porosity	Slow degradation, structural integrity		Abbott et al., 2016b
	SF	Mature unilocular cells	Porosity, surface topology	3D geometry		Abbott et al., 2018
	Collagen embedded SF	Human embryonic-derived stem cells	Porosity, surface topology	3D geometry		Wang et al., 2017
	SF	Subcutaneous adipose tissue	Porosity, surface topology, mechanical properties	3D geometry, structural integrity, perfusion		Abbott et al., 2015

## Tailoring Silk Biomaterials to Control Cellular Fate

### Biomaterial Surface Topology

**FIGURE 3:**
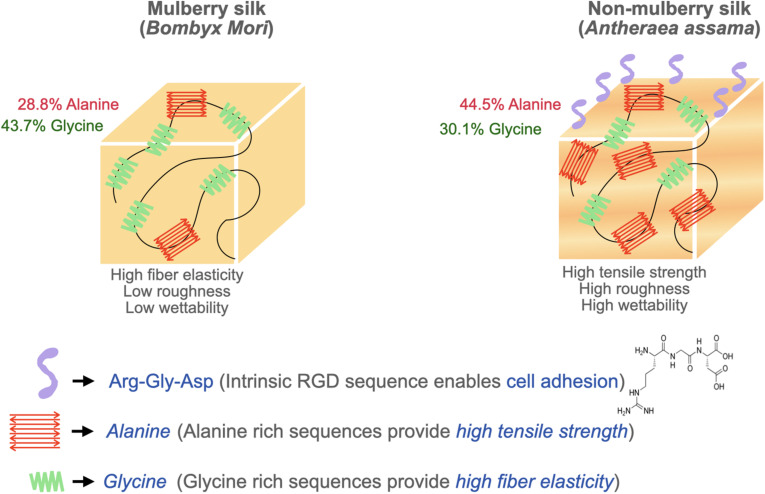
Biophysical attributes of mulberry (*B. mori*) and non-mulberry silk (*A. assama*).

Surface topography plays a crucial role in the regulation of cell adhesion (Guilak et al., 2009; Wong et al., 2013). Cell behaviors are regulated by nanotopography typically via the variation of cellular spatiotemporal dynamics and the sensing behaviors of intracellular mechanosensors (Chen et al., 2014). Silk proteins retrieved from different sources possess unique amino acids leading to varying chemistry, roughness, mechanical properties, and wettability (Figure 3). To compare the cell-substrate interface of different silk sources, vascular cells were cultured on mulberry *B. mori* and non-mulberry *A. assama* silk films (Gupta et al., 2019). After culturing vascular cells on a range of engineered silk films with different surface patterns, it was determined that *A. assama* films favored endothelial cell growth regardless of substrate alignment. In contrast, smooth muscle cells required unidirectional alignment to develop a contractile phenotype that was observed for both silk sources. This study highlights the important synergistic interaction between cell type, surface topography, and physicochemical properties of silk biomaterials for dictating cell fates (Gupta et al., 2019).

#### Substrate Alignment and Surface Patterning

Unlike flat surfaces, nanofibrous substrates can upregulate integrin expression to promote cell adhesions (Bottino et al., 2011). By harnessing substrate alignment at the nano- and micro-scale, silk materials can be designed to tailor cellular fates. For example, the desirability of silk fibers with a narrow distribution of widths offer comparable morphological cues from individual fibers to support collective cell development (Zhu et al., 2015). In a separate study, soft lithography was used to surface pattern SF to evaluate the effect of surface morphology on cell proliferation, orientation, and ECM alignment on corneal fibroblasts (Gil et al., 2010). Interestingly, the depth of the grooves was found to have greater impact on the cell orientation compared to the width (Patil et al., 2020). Patterning silk has also been harnessed to design “co-culture” systems that provide spatial control over homo- and hetero-typic cellular interactions at the micron level (Battiston et al., 2014). For instance, corneal stroma and corneal epithelium have been cocultured in one system using micropatterned silk films (Gosselin et al., 2018). Additionally, surface patterning can be utilized to regulate cellular proliferation. For instance, Schwann cells and PC12 cells proliferate more on aligned silk hydrogels. Additionally, the cells grow along the oriented layers, display elongated shapes, and have a significantly narrow angular distribution (Wang et al., 2019).

It should be noted that the extent and direction of influence of nano-patterning on cell proliferation varies with different cell types. Contradictory studies have shown that surface patterning can have opposite effects on endothelial cell proliferation (Lu et al., 2008; Moffa et al., 2014; Gupta et al., 2019). A plausible explanation for this discrepancy might suggest this phenomenon is related to the size, depth, and peak distance of grooves. Furthermore, when culturing smooth muscle cells on aligned silk films, a suppression of cell proliferation rate is typically observed, a feature observed during phenotype transition from synthetic (high proliferation index) to contractile (low proliferation index) (Beamish et al., 2010).

Substrate alignment also plays a crucial role in forming optimal niches for guiding stem cell differentiation toward neuronal and osteogenic lineages. For example, SF nanofibrous matrices with aligned structures were used to guide nerve cell regeneration (Dinis, 2014). In another study, secretion of nerve regeneration factors was observed on aligned silk-graphene composite hydrogels confirming the stimulating effect of aligned structures for stimulating a neuronal phenotype (Wang et al., 2019). Furthermore, another report investigated neuron differentiation and found that laminin-coated electrospun aligned SF mats showed an increase in neuron differentiation compared to the non-aligned control groups (Li et al., 2019). In another study, nanofiber-graphene composite scaffolds which not only induced cell neurites to arrange along the fiber direction, but also promoted the growth of cells with significant expression of neuronal marker β3-tubulin (Qing et al., 2018). Additionally, patterned SF films supported osteogenic differentiation along with lamellar cellular alignment and matrix deposition in a spatially controlled manner (Tien et al., 2012). Therefore, alignment and patterning of silk scaffolds can be considered to tune cellular differentiation.

#### Surface Roughness

The cellular response to silk’s surface roughness is controversial due to inconsistent methodologies across studies. One study indicated that surfaces with a moderate roughness (10–45 nm) with a nearly Brownian fractal dimension (∼2.5) promote maximum cell proliferation rates (Gentile et al., 2010). It has also been reported that cells experience higher proliferation only in a range of critical roughness (Anselme et al., 2000). Furthermore, it was reported a positive influence of surface roughness in the range of 1–7 nm on cell proliferation in the case of non-mulberry silk. However, a range of surface roughness beyond the “critical range” can result in lower cell proliferation in mulberry silk. This may be partly attributable to the absence of RGD sequences in mulberry silk (Mandal et al., 2010). On the other hand, other studies have reported insignificant differences in proliferation on varying surface roughness and eliminated its influence as a factor on cell proliferation during endothelial cell culture on silk films (Gupta et al., 2019). Such paradoxical behavior is attributable to the undefined aspects of surface roughness and requires more specific definitions that consider different nanostructure profiles, irregular features, grooves, and broadness of peaks.

### Surface Modifications

The origin of silk’s tunable biophysical properties and mechanical strength comes from its inherent ability to self-assemble into hydrophobic crystalline β-sheets; however, high β-sheet content materials result in inefficient cellular adhesion (Zhou et al., 2001; Cai et al., 2002). To combat this cellular interaction while maintaining desirable bulk material properties, surface modification of reactive amino acid residues on SF has been explored to achieve the surface attachment of small molecules, polymers, growth factors, cell binding ligands and ECM to improve cell adhesion and hydrophilic interactions on silk (Li et al., 2012).

SF has reactive carboxyl and amino groups in its side chains that can be conjugated to different functional groups. For example, plasma immersion ion implantation (PIII) treatments have been used to covalently bind proteins to the SF surface and improve cellular interactions (Kondyurin et al., 2018). Another simple surface modification technique involves plasma etching for grafting poly acrylic acid (pAAc) and poly hydroxyethylmethacrylate on regenerated SF films. The carboxyl functional groups on pAAc-grafted SFs can be further conjugated to other polymers and dyes like rhodamine. Furthermore, this technique can be used to tune the properties of SFs from low cell adhesion (unmodified SF) to high cell adhesion (pAAc–SF) and back to low cell adhesion (PEG–SF). Moreover, they achieved spatial control over the cellular adhesion property of the SF (Dhyani and Singh, 2014). A different study proposed a method to enhance bone matrix formation and hydroxyapatite (HA) mineralization by introducing carboxyl groups onto SF fibers. The abundance of polar and negatively charged groups also played a significant role in altering the protein assembly process and providing chemical handles for further modification (Zheng et al., 2016).

It has also been demonstrated that amine- and carboxyl- functionalized nanocomposite scaffolds can direct differentiation of human adipose derived stem cells toward osteogenic and chondrogenic lineages, respectively (Griffin et al., 2017). Silk films can be modified with carboxylic groups and phosphate groups using graft polymerization to control cellular differentiation (Patil and Singh, 2018). Silk films with carboxylic groups induced chondrogenic differentiation of human mesenchymal stem cell (hMSC), whereas those with phosphate groups induced osteogenic differentiation. Furthermore, grafting of these functional groups on silk simultaneously can provide spatiotemporal control over differentiation on the same surface (Patil et al., 2020).

Surface functionalization can also be used to mimic ECM specific signaling. Surface biofunctionalization with cell binding RGD peptides has been used to enhance cell proliferation through an integrin-mediated process (Rajendran et al., 2013; Gupta et al., 2019). Another ECM mimetic example is the decoration of SF films with integrin-binding laminin peptide motifs. These materials provide a rational design to mimic the functions of high molecular weight laminin proteins while also circumventing the high costs and stringent handling conditions that are linked with using whole laminin proteins (Manchineella et al., 2016; Li et al., 2019).

Surface modifications with inorganic compositions also have roles in regulating cellular processes. For example, a calcium phosphate-coated surface enhances cell proliferation, as compared to an uncoated one (Yang et al., 2010). Hydroxyapatite (HAp) functionalization is used to enhance the osteoconductive biological signals associated with osteogenesis and mineralization of stem cells (Ko et al., 2018). Cobalt (Co^2+^) has also been used as a dopant with silk owing to its unique ability to stimulate neovascularization. Co^2+^ when used with HA/SF scaffold, makes a great candidate for inducing angiogenesis and bone formation *in vitro* and *in vivo* (Fani et al., 2019). Therefore, inorganic elements can also be utilized in silk scaffolds to guide cellular differentiation.

### Silk Composites

SF can be amalgamated or cross-linked with proteins, polysaccharide, polymers, and other functional materials to make composites with the advantages of both materials (Patil et al., 2020). Cell adherence on silk is often hampered as a consequence of its hydrophobicity. For instance, silk fibers are stronger and stiffer while collagen fibers provide surface adhesion molecules. In this composite approach, the incorporation of silk is known to enhance the mechanical properties of collagen-silk composite fibers (compared to collagen alone) making it tunable for different applications. In silk-dominant collagen composites, after a delay in initial attachment, cells proliferate at a similar rate as that of cells on collagen-dominant composites (Zhu et al., 2015). In another study, enhanced cell adhesion and proliferation of nerve cells was observed on silk nanofibres through the introduction of exfoliated graphene sheets forming active cues to optimize cyto-responses (Wang et al., 2019). Genetic engineering is also utilized to functionally fuse ECM motifs to silk proteins. In one example, a motif from fibronectin was used to synthesize fibronectin-silk with the ability to self-assemble into networks of microfibers under physiological-like conditions to improve cell proliferation (Johansson et al., 2019). Therefore, depending on the application, composites can be considered to enhance cellular adhesion and proliferation.

Silk scaffolds can also be loaded with differentiation-inducing growth factors. For example, a silk microsphere/scaffold was developed with a concentration gradient to release multiple growth factors in a spatially controlled manner (Wang et al., 2009). In another study, SF nanohydroxyapatite scaffold were synthesized to enable sequential and sustained release of stromal cell derived factor-1 (Shen et al., 2016). Silk can also be used to reinforce nanoparticles. In one study, a water- dispersible HA nanoparticles was fabricated with SF nanofibers to create a scaffold with programmable sustained release of BMP-2 (Ding et al., 2016). Despite the widespread use of growth factors-loaded scaffolds, it is important to note that growth factors have short half-lives, lasting only a few minutes, and the doses that are reportedly efficient *in vitro* may not yield similar results *in vivo*.

### Mechanical Properties

Stiffer matrices induce tensional forces, causing the cell-matrix adhesion proteins to trigger a mechanotransductive pathway (Sridharan et al., 2009). This interaction between cellular and substrate mechanical modulus is a principal component of the reciprocal relation between cell and matrix (van Helvert et al., 2018). Silk demonstrates exceptional mechanical properties, including high tensile strength and extensibility, making it one of the toughest known materials (Shao and Vollrath, 2002; Becker et al., 2003). The exceptional strength of silkworm and spider silks, exceeding that of steel, arises from β-sheet nanocrystals that universally consist of highly conserved poly-(Gly-Ala) and poly-Ala domains. Despite the key molecular interactions in β-sheet nanocrystals being hydrogen bonds, size effects can be exploited to create bioinspired materials with tunable mechanical properties. In fact, silk has been tailored for applications as soft as the brain to as stiff as bone (Abbott et al., 2016a). However, reports suggest that cells spread better on stiffer silk substrates as opposed to those with low rigidity (Gupta et al., 2019). A plausible explanation for this behavior may be the imbalance between cell traction forces and corresponding ECM response, a crucial parameter for assembly of cell-matrix complexes and cell spreading. For instance, a study reported that the fine tuning of silk scaffold’s stiffness induces different endothelial migration and aggregation (Lu et al., 2019), suggesting a sensitive dependence of cell migration on mechanical cues.

SF is primarily utilized in its crystalline β-sheet form for tissue engineering applications. While higher β-sheet content reduces cellular adhesion and proliferation properties, it possesses superior mechanical strength (Manchineella et al., 2016). Non-mulberry silk films are mechanically stiffer and exhibited higher tensile modulus compared to mulberry silk films. The higher tensile strength and elongation of non-mulberry silk is attributable to the presence of distinct polyalanine stretches in its native structure, resulting in higher concentrations of antiparallel β-sheet structures compared to mulberry silk (Lefèvre et al., 2007). Therefore, silk source should be considered to tailor cellular outcomes. Physical interactions between cells and the stiffness and elasticity of the scaffold can influence stem cell behavior (Rowlands et al., 2008; Guilak et al., 2009). Stem cells have a tendency to differentiate into specific lineages when cultured on scaffolds with an elasticity similar to that of native tissue. The substrate’s elasticity affects the intracellular signaling through mechanotransducers such as Rho kinase and focal adhesion kinase which play a significant role in determining the stem cell lineage (Shih et al., 2011). This concept has been exploited to design tough anisotropic silk nanofiber hydrogels with different stiffnesses (52 and 120 kPa) for bone regeneration. This study demonstrated a higher expression of osteogenic genes on the stiffer hydrogels (120 kPa) revealing that a higher stiffness provides strong cues to control cell behaviors and osteogenic differentiation (Ding et al., 2020). Another study synthesized composite fibers using dragline silk with collagen at various ratios to examine the effect of mechanical properties on stem cell differentiation. The ultimate tensile strength and elasticity of the composite fibers increased with silk ratio while there was a slight reduction in stretchability (Ko et al., 2018). Their study concluded that the incorporation of silk proteins to collagen dramatically increased the matrix stability against excessive fiber swelling and shape deformation in cell culture medium. Matrices containing 15 wt% silk in collagen (CS15) and 30 wt% silk in collagen (CS30) were found to induce a level of neural differentiation comparable to that of pure collagen. In particular, CS15 matrix induced the highest extent of cell polarization and promoted the development of extended 1D neural filaments strictly in-line with the aligned fibers (Ko et al., 2018). In an effort to utilize different mechanical cues, another report used oxidized silk scaffolds to achieve a very high compressive modulus demonstrating the effect of matrix stiffness on fostering hMSC osteogenesis (35 times higher than their non-oxidized counterparts) (Zheng et al., 2016). The contrasting effects of different matrix stiffness and elasticity indicate the strong association of differentiation with mechanical properties.

### Porosity

Three dimensional porous silk scaffolds are used to provide structure and biomechanical cues for seeded cells until they are organized into a functional tissue (Zmora et al., 2002). Within these scaffolds, cell proliferation, migration, and differentiation are directly governed by size and porosity. To optimize pore microstructures and connectivity silk scaffolds can be fabricated with different porogens or freeze-dry regimes (Rockwood et al., 2011). Bigger pore sizes are generally linked to enhanced cell proliferation and migration (Murphy et al., 2010); however, 50–75 μm pores showed better cell proliferation than 75–100 μm pores in human primary dermal fibroblasts (Mandal and Kundu, 2009a). The effect of pore size on cellular fate is cell line specific. For bone marrow stromal cells expressing BMP7, a SF scaffold pore size between 100 and 300 μm resulted in enhanced cell proliferation and ECM production over smaller pore sizes (Zhang et al., 2010). For chondrocytes, however, the opposite has been observed with smaller SF scaffold pore sizes (90–250 μm) providing the best environment for adhesion and proliferation (Han et al., 2015). Therefore, porosity is a critical parameter that is cell type specific and should be optimized for each tissue engineered application.

## Conclusion

In summary, the biophysical properties of silk can be used to control cellular fate, including cellular adhesion, proliferation, migration, and differentiation. Beyond the inherent properties of silk, which can be patterned and aligned, the ability of silk to be combined with other functional materials or undergo relatively simple surface modifications can enhance or tune its biophysical influence on cells. Moreover, the exceptional mechanical properties of silk make it well-suited for a variety of tissue engineering applications. Finally, cellular behavior is critically regulated by silk type owing to a strong dependence on the availability of RGD sequences, hydrophilicity, and mechanical properties.

## Author Contributions

DK, MD, and RA performed the conception or design of the work, revised it critically for important intellectual content, provided approval for publication of the content, and agreed to be accountable for all aspects of the work in ensuring that questions related to the accuracy or integrity of any part of the work are appropriately investigated and resolved. DK drafted the work. All authors contributed to the article and approved the submitted version.

## Conflict of Interest

The authors declare that the research was conducted in the absence of any commercial or financial relationships that could be construed as a potential conflict of interest.
